# Deaths in jail: a retrospective analysis of autopsies performed at the Legal Medicine Unit of Pavia (1999-2022)

**DOI:** 10.3389/fpsyt.2024.1423325

**Published:** 2024-09-02

**Authors:** Giacomo Belli, Mateus E. Romão, Riccardo Lacquagni, Barbara Bertoglio, Andrea Bertolotti, Gaia Tamellini, Luca Morini, Gulnaz T. Javan, Ilaria Setti, Silvia D. Visonà

**Affiliations:** ^1^ Department of Public Health, Experimental and Forensic Medicine, University of Pavia, Pavia, Italy; ^2^ Unit of Applied Psychology, Department of Brain and Behavioural Sciences, University of Pavia, Pavia, Italy; ^3^ Department of Physical and Forensic Sciences, Alabama State University, Montgomery, AL, United States

**Keywords:** death in custody, detention regime, safety in prison, suicide prevention, inmates

## Abstract

**Introduction:**

The high rate of incarceration, now exceeding 11.5 million people worldwide, has raised concerns about the conditions within penal institutions, such as the consequences of incarceration on the person. This retrospective study aims to investigate the issue of death in custody, exploring the relationship between incarceration, health vulnerabilities, and death from the forensic pathologist’s point of view.

**Methods:**

We analyzed, from a forensic, clinical and toxicological perspective, 86 cases of deaths in detention facilities in North-Western Italy from 1999 to 2022.

**Results:**

The analysis has shown that suicide, mainly committed by hanging, plastic bag suffocation and butane intoxication, represents the leading type of violent death (52%), followed by accidental deaths (16%). On the other hand, cardiovascular diseases are the leading cause of natural deaths (42%), followed by infectious diseases (especially HIV-related).

**Discussion:**

The present study identifies the most frequent and critical situations and risk factors related to death in custody and the profile of the inmate who is at a higher risk of death, allowing to highlight the issues to be addressed from a public health point of view. On the whole, it calls for comprehensive reforms, aligned with international human rights standards, addressing mental and physical care gaps, improving correctional officers' education, and training, and focusing on rehabilitation with well-being and dignity.

## 1 Introduction

Currently, more than 11.5 million people are estimated to be held in penal institutions worldwide (1). Since the 1990s there has been a substantial growth in incarceration According to the database of the National Penitentiary Department of the Italian Ministry of Justice and ISTAT (Italian Institute of Statistics) data (2, 3), in Italy, there are approximately 56,196 inmates (55,831 men - 2,365 women), 96 inmates per 100,000 habitants, divided into 189 overcrowded facilities. Most of them are aged between 50 and 59 years old (n=10,662, 18,9%). On average, women are younger than men. Regarding nationality, most of the inmates are Italians (69%, 37091 people in 2021), followed by Moroccans (6% - 3291), Romanians (3,5% -1880), and Albanians (3,4% - 1820).

In terms of physical health, inmates are more vulnerable to infectious diseases, such as HIV, hepatitis B and C, and tuberculosis (4), and the incidence of chronic illnesses, such as cancer, cardiovascular disease, and respiratory problems, is greater in prison than in the general population (5). Moreover, a disproportionately higher prevalence of drug abuse (e.g., alcohol, opiates (including heroin), cocaine, methamphetamine, and marijuana) is detected, especially in women’s institutions.

Moreover, incarceration is a risk factor for a number of social, economic, and health issues (especially psychological and physical disorders). In addition, it determines changes in family structure, household disadvantage, and poorer mental health in children.

Among the psychological disorders, depression, bipolar, anxiety, stress, and psychosis are the most frequent in inmates. During detention, several environmental factors could have a negative influence on mental health, such as confinement and social restriction, violence and safety concerns, limited access to physicians and psychologists, stigma and lack of rehabilitation and reintegration programs, and drug abuse (6). Altogether, these conditions might have serious consequences, such as death in custody.

The term “death in custody” refers to the death of an individual under the responsibility of law enforcement, encompassing the arrest, transportation to detention facilities, and the ensuing period of confinement (7).

Deaths that occur within penal institutions can be generally classified, according to the manner of death, as natural and violent deaths. Natural deaths in prison are mostly represented by cardiovascular disorders, and infectious diseases. On the contrary, violent deaths in jail include (but are not limited to) homicides, suicides, accidents, drug-related deaths, and fatalities resulting from punitive interventions such as torture and restraint (8).

Based on Italian National Statistical Institute (2023) (9), from 2000 to 2023 there were approximately 3,637 deaths in Italian penal institutions, which represents a mean rate of 158,1 deaths per year (the highest rate was recorded in 2011 with 186 cases and the lowest rate in 2016 with 115 deaths). Addressing the problem of death in custody from a medico legal point of view is of great importance, not only to bring about issues related to human rights and justice within the criminal justice system, but also to identify new legal and public health policies for the promotion of health in jail (10). Nevertheless, it is relevant to highlight that every individual, regardless of their legal status, has a right to a life in dignity and access to health care, as stated by the Italian Constitution (art.32) and guaranteed by the legislation on detention (11, 12).

Besides, it is not possible to understand the strict liability of the Detention Facilities. In cases of death in prison, a personalized evaluation is usually carried out to identify any responsibility. The Italian Supreme Court often recognized the health care workers as responsible, especially for an inadequate indication for the supervision of subjects with greater risk of performing self-harming behaviors or suicide, as well as for a failure to diagnose and prevent infectious and/or chronic diseases (13). On these bases, death in custody represents a significant public health issue (14). The present study aims to analyze retrospectively a series of deaths in detention facilities, in order to understand more clearly the phenomenon of death in jail, focusing on the biological and behavioral profile of the deceased subjects.

## 2 Materials and methods

Cases of death in detention facilities were selected from a dataset of 8,139 autopsies, both forensic and clinical, which were performed from 1999 to 2022 at the Unit of Legal Medicine and Forensic Sciences of Pavia University, Italy. For each case, demographical data (i.e., age at death, sex, and citizenship), social status (previous employment and marital status), clinical records (clinical data, with a focus on biological, mental, or neurological illnesses, such as the history of drug abuse), and forensic assessments (i.e., time, circumstances, and manner and method of death) were collected. According to the manner of death, the selected cases were classified as violent (i.e., homicide, suicide, accident), or natural. For cases of suicide by hanging, information on the means used was recorded. In addition, the existence of suicide notes and a history of previous self-harming, suicide ideation, and attempts were also considered. Where the autoptic examination was integrated with toxicological analyses, data on drug and therapy intake were also considered and analyzed. The above listed variables have been extracted from the informatic archive of the Department. To ensure the subject’s privacy, all data were anonymized.

Statistical analyses were performed using the R Studio Software (version 2023.09.0 + 463) (RStudio Team (2015). RStudio: Integrated Development for R. RStudio, Inc., Boston, http://www.rstudio.com/) (15). Specifically, Chi-squared test and one-way analysis of variance (ANOVA) were carried out. Statistical significance was assessed when the p-value was lower than 0.05.

Two cases were excluded from the statistical analyses (the only female subject and the male homicide victim) to avoid the introduction of possible bias.

## 3 Results

On the whole, 86 cases of death in detention facilities located in North-Western Italy were examined. In all cases, a judicial autopsy was requested by the Prosecutor to define the time, causes, and manner of death. The series was mainly composed of men (98.8%, 85 males and only one female), Italian citizenship (71%, 61 individuals), aged on average 41.0 ± 11.6 years (min-max: 22-81 years old). Since in the series only one female was selected according to the selection criteria, she was excluded. **Table 1** summarizes the personal and social information for the male sample. Most of the deaths were violent (69%), and specifically, suicidal events were the most numerous (52%), followed by accidental deaths (16%), and homicides (1%). Natural deaths occurred in 31% of the cases. Since homicides are represented by only one case, they were not included in the following analyses. A similar mortality rate was detected between Italian and foreign inmates for the three manners of death. Suicides represented half of the cases in both Italian (51%) and foreign inmates (56%), while the remaining deaths were ascribed to accidental (in 24% of Italians and 34% of foreigners) and natural causes (15% and 20%), (**Table 1**). These observations were corroborated by statistical analyses (Chi-squared test), that showed no significant difference between Italian and foreigners (p-values > 0.05). When considering age at death, persons who died from natural causes were older (mean age: 48.8, SD: 11.8 yrs) if compared to those who died from accidental causes (mean age: 40.4 yrs, and SD: 11.1 yrs.) or suicide (mean age: 36.2 yrs, SD: 9.1 yrs). This pattern was also seen in the group of Italian prisoners, but not in the group of foreigners (**Table 1**). Statistical test (ANOVA) confirmed this pattern, showing a significant difference among the three manners of death (p-value < 0.001). Specifically, according to the age-at-death, statistical differences (comparing Italians with foreigners) were observed between natural and accidental deaths (p-value < 0.05) and between natural and suicide events (p-value < 0.001). However, no significant difference was found between accidental and suicidal deaths (p-value> 0.05) according to the Tukey HSD test.

**Table 1:** Male sample data. Details of the biological profile and social status.

Manner of death	Citizenship	N	Mean age (± SD)	Marital status					Previous job		
				Single	Married	Divorced	Widowers	NA	Yes	No	NA
**Accident**	Italy	9 (15%)	42.2 (± 13.4)	5	2	0	1	1	4	3	2
	Foreign	5 (20%)	37.2 (± 4.9)	2	2	0	0	1	3	0	2
	**All**	**14** **(17%)**	**40.4** (± **11.1)**	**7**	**4**	**0**	**1**	**2**	**7**	**3**	**4**
**Natural**	Italy	20 (34%)	51.6 (± 11.4)	10	3	0	1	6	5	2	13
	Foreign	6 (24%)	39.7 (± 8.6)	3	1	0	0	2	2	0	4
	**All**	**26** **(31%)**	**48.8** (± **11.8)**	**13**	**4**	**0**	**1**	**8**	**7**	**2**	**17**
**Suicide**	Italy	30 (51%)	38.3 (± 8.7)	14	6	1	0	9	9	2	19
	Foreign	14 (56%)	31.8 (± 8.5)	7	0	0	0	7	1	0	13
	**All**	**44** **(52%)**	**36.2** (± **9.1)**	**21**	**6**	**1**	**0**	**16**	**10**	**2**	**32**

In bold, the data presented pertains to the total population of incarcerated individuals analyzed globally, regardless of whether they are of Italian or foreign origin.

Data on previous employment and marital status were available for only 36.5% of Italians and 69.4% of foreigners, respectively. The workers were more than unemployed people, and inmates were mainly single, followed by married, and divorced/widowers. Where the information was available, no differences in the marital status were detected according to the manner of death.

Further data are reported below according to the manner of deaths.

### 3.1 Traumatic deaths

#### 3.1.1 Suicide

(44 cases): the main cause of death is acute mechanical asphyxia by hanging (31 subjects), followed by complex suicide through butane intoxication with a plastic bag (6 subjects), butane intoxication (3 subjects), plastic bag suffocation (2 subjects), and drug intoxication (2 subjects). Most suicides by hanging were carried out using bed sheets (10 cases), bathrobe rope (5 cases), belt (2 cases), and shoelaces (2 cases), while sporadic cases with other strangulation tools (e.g., shirt, fabric noose, rope, elastic, scarf, and t-shirt). Little information was retrieved about previous suicide attempts, ideation, and self-harming: data were available for only 20% of the cases (9 subjects), and specifically, previous suicide attempts were reported in 7 out of 9 cases, and in 2 cases a suicide note was written. Considering the pathological conditions, data could be collected from a few cases only: 7 inmates had a history of psychiatric disorder (mostly depression) and 13 were drug abusers. In two cases, the subjects were both drug addicted and psychiatric patients.

Toxicological investigations were performed in 24 cases, highlighting especially a therapeutic use of pharmaceutical drugs (12 cases), while only 3 cases involved abuse of unlawful drugs (opioid and cocaine), and the remaining showed negative results (8 cases). No evidence of alcohol consumption was identified.

#### 3.1.2 Accidental deaths

(14 cases): in this group, most deaths occurred due to acute intoxication by drugs (cocaine or heroin, 12 cases), and only two cases due to blunt force trauma. Six inmates were drug abusers and in 2 cases the subjects were both drug abusers and psychiatric patients. The toxicological investigations, which were conducted in 12 cases, highlighted in all of them either the use of illegal substances or an unintentional overdose of therapies. For six individuals, the alcohol test gave positive results.

#### 3.1.3 Homicides

(1 case): In our data set, only one murder was identified. The victim, a 57-years-old Italian man, was assaulted during the night by his cellmate. The victim was first struck with a blunt object, causing significant head trauma, and then murdered by strangulation using a belt.

### 3.2 Natural deaths (26 cases)

**Figure 1:**
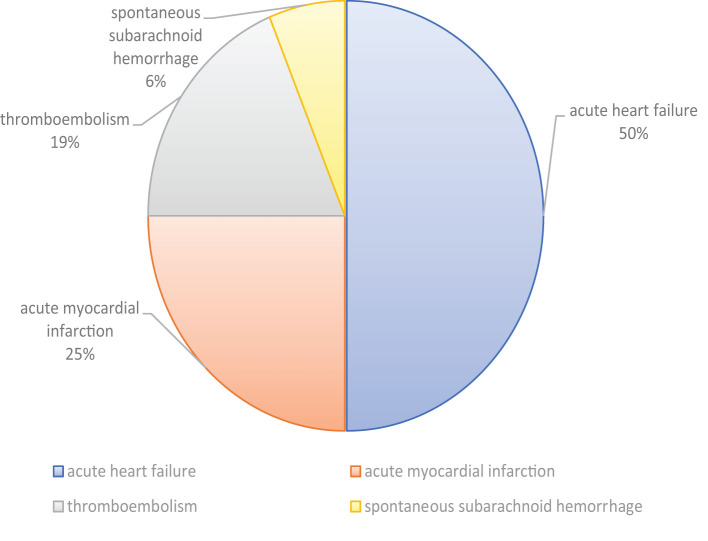
Causes of death in the group of natural deaths due to cardiovascular pathologies.

In this group, cardiovascular diseases are the most frequent, causing death in 17 subjects (8 cases of acute heart failure in bi-three-vessel coronosclerosis; 4 cases of acute myocardial infarction; 3 cases of thromboembolism; 1 case of spontaneous subarachnoid hemorrhage) (**Figure 1**), followed by infectious diseases (4 subjects). In 23 cases, a positive medical history was detected. In particular, 3 inmates were HIV positive, 9 inmates suffered from cardiovascular disorders, and 7 subjects from psychiatric conditions/disorders. One subject was a drug abuser. The toxicological investigations were conducted in 10 cases, resulting in 6 negative and 4 positive tests. In the last, a pharmacological therapy was discovered. In 2 cases, alcohol consumption was detected.

## 4 Discussion

In this study we analyzed 86 cases of death in custody in which a forensic autopsy has been performed, in order to better understand what are the most frequent situations and which is the profile of the inmate at higher risk of violent or natural death. This study aimed to identify areas of public health intervention to prevent deaths in custody.

Our results are in agreement with the literature data, which highlighted, in the incarcerated population, a higher probability of suffer from chronic cardiovascular and metabolic diseases (heart diseases, stroke, and diabetes), infectious diseases (HIV/AIDS, tuberculosis, and hepatitis), psychological conditions (PTSD, depression, and anxiety) and a higher risk of suicide (16). According to Daniel et al. (17), the high incidence of diseases and the high mortality rate in inmates are mostly due to maltreatment and negligence: for example, substance abuse, no access to physicians and psychotherapists, social isolation, and a hostile environment.

In agreement with the literature, in our series, suicide is the main cause of death, accounting for the 52% of the cases. Literature studies showed in the detained population a suicide rate 2-3 times greater than that observed in the general population (18). Particularly, Castelpietro and colleagues (2018) identified a mean rate for suicide and suicide attempts equal to 1.12 and 15 per 1000 inmates in Triveneto area (North-Eastern Italy) (19). As expected, the suicide rate among prisoners, considering gender, is higher in the male population. This observation aligns with the general epidemiology of suicide, which shows a clear predominance of male victims in Italy, and the fact that there is a significant disproportion between males and females within the prisons (women are, indeed, less than 5% of prisoners) (2–20): for example, in 2021, according to ISTAT, there were 3,870 deaths in Italy, of which 3,036 were male and 834 were female, with a ratio of nearly 1:4. During the same year, the Antigone Association (an Organization focused on prisoners’ rights) reported 57 suicides in prison, all male subjects (9–21)..

A possible explanation for the high incidence of suicide is the prison environment, which is harmful to human beings due to the architecture and the punitive measures adopted. According to Skinner (1948), there are two ways to punish a person: introducing the person to an unfavorable stimulus (i.e., incarceration), and removing something considered pleasant (i.e., social relations) (22). Several reasons and scientific data evaluate the side effects of punishment on human cognition and behavior, mainly if for long periods. From a behavioral point of view, punishment produces an unpleasant emotional state, aggressive behavior, impairments in social relations, avoidance behavior (23–25). From a neuropsychological point of view, there is a cognitive decline, such as executive functioning, cognitive control, emotion regulation, recognition, attention, and learning (26). In other words, incarceration affects extremely important activities of executive function, such as planning, working memory, taking initiative, and impulse control (27). These functions are essential for living with dignity, and with a proper house and job (28). In addition, some studies showed that long isolation periods with minimal stimulation led to poorer mental health, as well as intense feelings of anger, frustration, and anxiety, which could result in suicide or drug abuse to relieve the long hours of tedium (6).

Moreover, in a meta-analysis (29), Franklin and colleagues (2017) highlighted the main risk factors for suicidal thoughts and behaviors, and among them incarceration, psychological disorders (e.g., depression, bipolar, and substance abuse), social isolation, impulsivity, childhood abuse, and trauma. In addition, epidemiological data suggests aggression, hopelessness, social disengagement, age, gender, distress, unemployment, loss of social support, and neurohumoral activity as some of the risk factors for suicide (30).

According to the European Monitoring Centre for Drugs and Drug Addiction (2022), drug consumption in prison is a public health and safety risk to the inmates and correctional officers, even though several drug-related interventions exist to prevent the consumption and the number of deaths due to drug abuse is still significant (31). The present study showed the real consumption of illegal drugs (12 cases of cocaine and at least 2 cases of heroin consumption) and alcohol (15 cases were positive to the alcohol test). Security management to avoid drugs within detention facilities is a hard task, as already stated by O’Hagan & Hardwick (2017), who suggested that visitors, new or returning prisoners, post, and corrupt prison staff are usually responsible for transporting drugs and illegal contraband inside prison (32).

In this study, cardiological conditions were the main cause of natural deaths (42%). Similar results were observed by Gentile et al. (2021) (Italy; 51%), Fazel et al. (2006) (UK; 42%), and Unal et al. (2016) (Turkey; 42%) (33–35). Although the risk factors for cardiovascular accidents are well known today, most deaths related to cardiovascular diseases occurred in inmates during transport to the hospital, or in the first 12 hours after hospitalization (81%). Moreover, in some cases, inmates died due to a worsening of chronic disease, already well-known in the clinical records. In agreement with the literature, an explanation for this phenomenon could lie in the lack of training of the prison operators (law enforcement), who are not able to recognize cardiovascular symptoms, with possible delays in the diagnostic-therapeutic process (36), especially in overcrowded contexts, where access to first medical care is limited by the number of users. Many cardiac deaths in prison, therefore, could be avoidable, especially considering the link with lifestyle, such as diet, physical activity, and drug consumption (e.g., cocaine, heroin, or even tobacco). All those aspects of life could be controlled and improved, even in a prison context (7).

Infectious diseases were significant as well, as reported in the literature. In fact, the analyses highlighted infectious diseases as the second cause of death for natural causes (mostly associated with HIV infection/immunodeficiency). It is well-known that the spread of infectious diseases is inherent in living in confined environments, especially when it is overcrowded. Therefore, aware of this fact, attention within the detention facilities should be more accurate, in agreement with the specific epidemiology guidelines.

### 4.1 Limitations

The present study has some limitations. The sample size is not representative of the total phenomenon of prison fatalities in a given geographical area. Prosecutors sometimes archive some cases based on circumstantial evidence. However, in the vast majority of death in custody the autopsy is performed.

## 5 Conclusions

The phenomenon of death in prison is a serious public health problem. The Italian Constitution (1947), Article 27 (37), establishes the re-educational purpose of the sentence in correctional settings, and Article 32, the right to health of all individuals. Even though the Italian Penal Code and the Legislation of the Prison Act and the Code of Criminal Procedure state the government’s duties with the incarcerated population, scientific evidence has shown a different reality. For instance, Italy was convicted more than once, by the European Court of Justice for the violation of Human Rights for inhumane and degrading treatment, and currently, after the COVID-19 pandemic, the suicide rate among the incarcerated population in Italy has increased brutally, 15.4 per 10,000 inmates (38). According to Mulgrew (2023), death in custody is completely preventable and easy to state and substantiate (39). This study has point out some scenarios that require action in order to prevent deaths in custody. We can summarize the main areas of interventions as follows.

1. Healthcare accessibility, regular medical screenings, and assessments to detect existing conditions and prevent potential deaths. 2. Staff training and education for prison operators to allow them to promptly identify and respond to emergencies (considering the necessity of addressing the current scarcity of prison police officers - the Ministry of Justice reports that as of 2024, 16% of the organizational chart’s seats remain unfilled -). 3. Mental health initiatives and access to psychologists, as well as the implementation of mental health programs for inmates, to decrease psychological disorders, suicide rates, self-harm-related fatalities, and coping strategies for emotional valence and arousal. 4. Guidelines about the use of force or other forms of punishment in order to reduce situations that escalate and result in fatalities or deaths 5. Addressing overcrowding and improving living conditions to mitigate health issues reducing stress and violence. 6. Rehabilitation Programs and evidence-based programs to give the inmates the repertoire to have skills for a life with dignity after their release. 7. post-release support, the support to the transition into society through healthcare, mental care, and employment opportunities to decrease post-release fatalities and recidivism rates (40–43).

## Data Availability

Data collecting, such as sampling, toxicological examinations and forensic analysis were ordered by the Prosecutor and therefore related to Penal cases. Requests to access the datasets should be directed to giacomo.belli01@universitadipavia.it.
